# Is WALANT Really Necessary in Outpatient Surgery?

**DOI:** 10.3390/jpm15010001

**Published:** 2024-12-24

**Authors:** Guido Bocchino, Silvia Pietramala, Giacomo Capece, Leopoldo Arioli, Alessio Greco, Stella La Rocca, Lorenzo Rocchi, Camillo Fulchignoni

**Affiliations:** 1 Department of Orthopaedics and Traumatology, Fondazione Policlinico A. Gemelli IRCCS, 00168 Rome, Italy; guido.bocchino01@icatt.it (G.B.); giacomo.capece01@icatt.it (G.C.); grecoalessio9@gmail.com (A.G.); stella.larocca01@icatt.it (S.L.R.); 2 Department of Orthopaedics and Traumatology, Universita’ Cattolica del Sacro Cuore, Largo Agostino Gemelli 8, 00168 Rome, Italy; lorenzo.rocchi@policlinicogemelli.it (L.R.); camillo.fulchignoni@gmail.com (C.F.); 3 Orthopaedics and Hand Surgery Unit, IRCSS Policlinico Gemelli, 00168 Rome, Italy; leopoldo.arioli@guest.policlinicogemelli.it

**Keywords:** WALANT, outpatient surgery, hand surgery, tourniquet

## Abstract

**Introduction**: The Wide Awake Local Anesthesia No Tourniquet (WALANT) technique has revolutionized outpatient hand surgery, enabling procedures such as carpal tunnel release and trigger finger release without a tourniquet. Its benefits include patient cooperation during surgery, especially for tendon repairs. However, WALANT has limitations, including a steep learning curve, longer operative preparation time, and risks such as digital ischemia and adrenaline-induced cardiac ischemia. This study evaluates the safety and effectiveness of local anesthesia with a tourniquet for short-duration outpatient hand surgeries. **Materials and Methods**: This case series included 300 patients undergoing carpal tunnel or trigger finger release between February 2023 and March 2024. Local anesthesia with lidocaine was administered, and a tourniquet was applied to the proximal arm. Demographic data, operative time, and pain levels during tourniquet use (measured by VAS) were recorded. **Results**: The average surgical time was 12 min. Most procedures involved carpal tunnel release. The average VAS pain score was 3.73, with older patients and longer surgeries reporting higher discomfort. Tourniquet release was required in only 1% of cases due to discomfort. **Conclusions**: For short outpatient hand surgeries, local anesthesia with a tourniquet is a safe, effective alternative to WALANT, challenging its routine use and highlighting the need for tailored anesthetic approaches.

## 1. Introduction

Carpal tunnel syndrome is one of the most common conditions described in hand surgery, followed by trigger finger and De Quervain’s disease [1].

Carpal tunnel syndrome is a common condition that results from the compression of the median nerve as it passes through the carpal tunnel, a narrow canal in the wrist. The carpal bones, which form a concave arch, create the tunnel’s floor and lateral walls, whereas the carpal tunnel’s roof is formed by the transverse carpal ligament (also known as the flexor retinaculum). This strong fibrous band stretches across the carpal arch, covering and enclosing the tunnel, and is a key structure in maintaining its integrity. The median nerve is responsible for sensation in the thumb, index, middle fingers, and radial side of the ring finger, as well as motor functions in some of the small muscles of the hand. The symptoms of CTS often include numbness, tingling, pain, and weakness in the hand, which are typically more pronounced at night or after repetitive activities like typing, using tools, or gripping objects. In advanced cases, it may lead to difficulty performing fine motor tasks and muscle atrophy in the base of the thumb. The condition is associated with repetitive motion, wrist injuries, pregnancy, and systemic illnesses such as diabetes, arthritis, and hypothyroidism. Diagnosis involves clinical evaluation using tests like Tinel’s sign, Phalen’s maneuver, and sometimes nerve conduction studies to confirm nerve compression. Initial treatment may include wrist splints, activity modification, and anti-inflammatory medications. In cases where symptoms persist or worsen, corticosteroid injections or carpal tunnel release surgery may be necessary to alleviate pressure on the nerve and restore hand function [2].

Trigger finger, medically known as stenosing tenosynovitis, is a condition where one or more fingers lock in a bent position and may suddenly snap straight, mimicking pulling and releasing a trigger. This happens when the flexor tendon becomes inflamed or develops nodules, making it difficult to glide smoothly through its sheath. Over time, this inflammation can cause the sheath to thicken, narrowing the space through which the tendon moves. Trigger finger is most common in individuals whose work or hobbies involve repetitive gripping motions, such as musicians, industrial workers, or those with frequent tool use. It is also more prevalent in people with conditions like diabetes, rheumatoid arthritis, or gout. Symptoms typically include stiffness, tenderness, a catching or popping sensation, and pain at the base of the affected finger, particularly in the morning or after prolonged use. Initial treatments involve rest, splinting, and anti-inflammatory medications. Steroid injections are also effective in reducing inflammation. If conservative treatments fail, a minor surgical procedure may be performed to release the tendon sheath and restore smooth finger movement [3].

De Quervain tenosynovitis is a painful condition that affects the tendons located on the thumb side of the wrist in the first extensor tendon compartment, specifically the abductor pollicis longus and extensor pollicis brevis. These tendons are encased in a sheath that facilitates smooth gliding. Repetitive motions such as lifting, gripping, or twisting, particularly in activities like lifting children, playing racquet sports, or texting, can lead to irritation and inflammation of the tendons and their sheath. Symptoms of De Quervain tenosynovitis include pain and swelling at the base of the thumb, tenderness along the affected tendons, and difficulty gripping or pinching objects. The pain may worsen with thumb and wrist movements, such as forming a fist or turning a doorknob. Diagnosis often involves a clinical examination, including Finkelstein’s test, which provokes pain when the thumb is tucked into the palm and the wrist is moved. Treatment typically begins with conservative approaches, such as rest, thumb spica splints, and anti-inflammatory medications. Corticosteroid injections may provide significant relief by reducing inflammation. In severe or persistent cases, surgical intervention may be necessary to release the sheath and alleviate pressure on the tendons [4].

Achieving a bloodless surgical field when treating these conditions surgically is crucial to clearly identifying anatomical structures and minimizing the risk of iatrogenic injuries [5].

Typically, a transient ischemia is obtained by placing a tourniquet or pneumatic sleeve at the upper third of the arm, and it is kept during the whole surgical period. However, the tourniquet may cause significant pain, discomfort, and poor tolerance when employing local anesthesia [6].

The Wide Local Anesthesia No Tourniquet (WALANT) technique, as described by Lalonde [7], is a type of local anesthesia which combines lidocaine and epinephrine to provide both anesthesia and vasoconstriction, which reduces bleeding during surgical procedures. The local anesthetic should be administered approximately 30 to 45 min before the procedure to allow adequate time for the anesthetic effect to set in and for the vasoconstrictive properties of epinephrine to take effect. This preemptive approach helps ensure optimal surgical conditions as it allows for both pain control and a clear surgical field [8].

The increasing popularity gained by this technique in the literature, combined with the possibility of avoiding tourniquet use, has created a debate around the best anesthesia technique for outpatients surgeries [9]. WALANT has already been affirmed as a reliable and useful tool in hand surgery, particularly in tendon injury repair when the patient’s cooperation is crucial to determining the correct tension of the suture, which, purportedly, is not possible when brachial plexus anesthesia is performed, despite no scientific evidence supporting this claim [10]. Moreover, using a tourniquet decreases the muscle strength of the hand and does not permit the surgeon to evaluate suture-appropriate tension. Additionally, WALANT is particularly advantageous in procedures requiring the patient’s active participation, such as tendon repairs, even though there is no definitive evidence supporting the benefits of this active involvement [11]. Despite the great enthusiasm which came along with WALANT, there are potential risks related to peripheral epinephrine injection, particularly in certain patient populations [12]. The most common adverse reaction associated with WALANT is fainting secondary to a vasovagal response [13]. In older adult patients with compromised circulation or rheumatic disorders, epinephrine-induced vasoconstriction can also exacerbate ischemic complications, increasing the likelihood of adverse outcomes such as digital ischemia. The risks of ischemic complications need to be weighed against the benefits of WALANT, particularly in populations with vulnerable circulatory systems [14].

This technique gained significant popularity during the COVID-19 pandemic, primarily because it does not require the presence of anesthesiologists, who were often occupied with intensive care duties [15]. Furthermore, this local anesthetic technique provides lower risk of viral dissemination because it does require ventilation; it requires simpler operating rooms, minimum number of professionals, has lower material expenditure, and consequently, lower hospital waste production [16].

With this premise, the main advantages of WALANT, as described in the literature, besides the absence of a tourniquet, include savings in time and costs, as well as full patient cooperation [4,5]. However, in most clinics, outpatient surgeries take place in precise settings where patients arrive just before the procedure, making it challenging to perform WALANT in a timely manner. Furthermore, the level of cooperation achieved with local anesthesia and tourniquet (LA-T) is sufficient for this type of procedure. The only potential advantage of WALANT might be the absence of a tourniquet. The objective of this study is to demonstrate that local anesthesia with a tourniquet is well-tolerated by patients undergoing short-duration outpatient hand surgeries, highlighting its safety and effectiveness for these procedures.

## 2. Materials and Methods

### 2.1. Study Design

Ethical review and approval were waived for this study by the Review Board of our orthopedic and traumatology institute due to this retrospective, observational study being conducted in accordance with the PROCESS guidelines. This study observed national ethical standards and the Declaration of Helsinki. Written informed consent for surgical and clinical data collection for scientific purposes was obtained from all patients upon admission and before surgery according to the institutional protocol. At the Hand Surgery Department of Policlinico Gemelli, we conducted a case series involving 300 consecutive patients who underwent outpatient surgical procedures for carpal tunnel syndrome, trigger finger, or De Quervain’s disease using local anesthesia with a tourniquet (LA-T) between February 2023 and March 2024. This study aimed to evaluate the outcomes of these procedures by analyzing demographic data, operative times, procedure types, and intraoperative pain associated with tourniquet use.

The patients were operated on by experienced hand surgeons using a consistent surgical approach, with the choice of local anesthesia and tourniquet as the standard practice.

The inclusion criteria for the study were patients diagnosed with carpal tunnel syndrome, trigger finger, or De Quervain’s disease, who required surgical intervention and were suitable for outpatient procedures using local anesthesia with a tourniquet. We included only those patients who did not have any concomitant injuries or conditions that would necessitate the use of spinal or general anesthesia.

The exclusion criteria involved patients with concurrent injuries or conditions that required more complex anesthesia, such as spinal or general, as well as those requiring additional surgical interventions such as synovial cyst removal, synovectomy, or tenolysis, which could excessively prolong the surgery and potentially skew the statistical analysis. Patients with severe comorbidities or circulatory disorders that could complicate the use of a tourniquet, as well as those with age-related or other specific factors that rendered the procedure inappropriate for local anesthesia, were also excluded from the study.

Demographic data, operative times, the type of surgical intervention, and the intraoperative pain associated with the tourniquet were systematically recorded and analyzed. Intraoperative pain was assessed using the Visual Analog Scale (VAS) score to evaluate the discomfort caused by the tourniquet.

After data collection, patients were divided into groups according to age and surgery duration.

Additionally, at the follow-up visit for stitches removal two weeks postoperatively, we collected data on the skin condition of the surgical wound to assess healing.

All patients received detailed information about the procedure, including the use of the tourniquet, and signed an informed consent form prior to surgery.

### 2.2. Surgical Technique

The surgery was performed according to the standard technique. No endoscopic nor eco-guided carpal tunnel or trigger finger release was included, as those techniques are not routinely executed in our center. Local anesthesia was administered on the operating table after the surgical field was set up. Transient ischemia started right before surgical incision.

For carpal tunnel release, a longitudinal or transverse incision was made over the carpal tunnel, and the transverse carpal ligament was carefully divided to decompress the median nerve. For trigger finger release, a small incision was made at the level of the affected pulley, and the A1 pulley was divided to allow for smooth tendon gliding. In cases of De Quervain’s tenosynovitis, an incision was made over the first dorsal compartment, and the extensor retinaculum was released to relieve tendon entrapment. Throughout the procedure, the tourniquet maintained a clear surgical field by minimizing blood flow to the area. After completing the surgical intervention, the tourniquet was deflated, and hemostasis was confirmed. The wound was then closed with absorbable sutures, and a sterile dressing was applied.

Postoperative care was standardized across all patients, with a postoperative analgesic regimen consisting of non-steroidal anti-inflammatory drugs (NSAIDs), as needed, based on the patient’s level of discomfort. The duration of analgesic use was determined by each patient’s individual needs.

Suture removal was performed in an outpatient setting 15 days post-surgery.

### 2.3. Intraoperative and Outcome Assessment

Intraoperative pain was defined as pain perceived by the patient during the surgery and pain related to the tourniquet in the regional anesthesia group. The pain was assessed using the Visual Analog Scale (VAS).

Complications were assessed 0 and 15 days after surgery and included the presence of a hematoma, infection, suture dehiscence, or skin necrosis.

### 2.4. Statistical Analysis

Statistical analysis was carried out on Microsoft Excel (2019). Average VAS scores in different groups are presented as means and standard deviation. Relationships between VAS score and patient’s age, as well as surgery duration, are expressed through t-student test with a confidence level α= 0.05.

## 3. Results

### 3.1. Demographics

Demographic data analysis revealed a population with a mean age of 64.02 years, comprising 37% males and 63% females. Patients were divided according to age (under 75 years old vs. over 75 years old; and under 80 years old vs. over 80 years old). As many as 75% of patients (225) were under the age of 75 years, while only 14.6% of patients were over 80 years old [Table 1]. Common comorbidities were overlapping in different groups. Despite hypertension being slightly more common in the over 75 years old group (58% vs. 47%), this correlation was not statistically significant (*p*-value > 0.05). As regards diabetes and hypercholesterolemia, they accounted for about 35% and 20% in both groups, respectively.

### 3.2. Type of Intervention

In our study, we examined 128 cases of carpal tunnel syndrome, 95 cases of trigger finger, and 77 cases of De Quervain’s tenosynovitis. 

### 3.3. Time of Intervention

The mean duration was calculated to be 12 min (range: 8–18). No significant differences were observed related to sex or age. However, the mean duration for carpal tunnel syndrome procedures was slightly longer compared to the other two procedures, with a value of 13.2 min (range 10–18).

The cases with longer durations often involved combined surgeries, such as trigger finger release in two fingers or a combination of trigger finger release and carpal tunnel syndrome surgery (Table 2).

Patients were divided into groups according to surgery duration; 82% of surgeries had a duration under 13 min, while only 7% lasted more than 15 s.

### 3.4. Tourniquet-Related Pain

**Figure 1:**
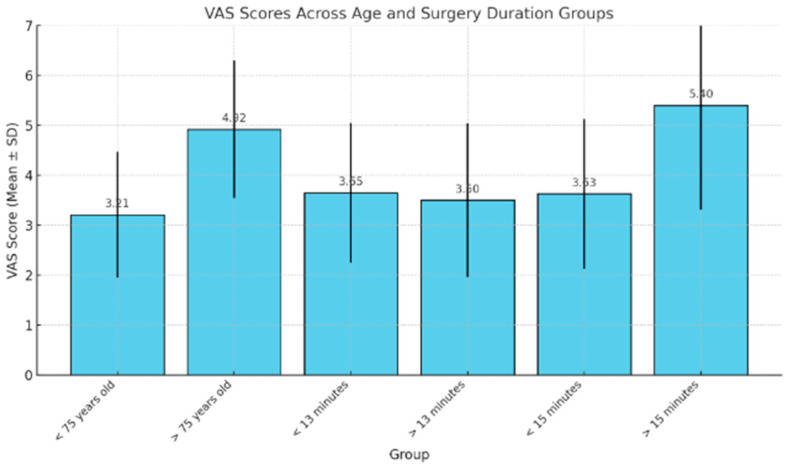
Graphic showing relationship between VAS score and age and surgery duration.

The mean VAS score related to the tourniquet was 3.73 (range: 1–10). No differences in pain levels were observed based on sex; however, discomfort related to the tourniquet was found to be related to ischemia time, with higher values in longer procedures. In particular, the relationship between VAS score and surgery duration turned out to be statistically significant only for procedures longer than 15 min. Higher VAS scores were noted in older patients, with an average score of 4.9 (range 2–8) in those over 75 years of age. VAS scores were independent of the procedures performed. Interestingly, there was no correlation between tourniquet-related pain and tourniquet pressure, suggesting that discomfort is related to duration rather than pressure (Table 3, Figure 1).

Notably, in only three cases was it necessary to release the tourniquet before the end of the procedure due to patient discomfort, and we were compelled to continue the surgical procedure while maintaining constant hemostasis and performing continuous washes with saline solution to ensure adequate visibility.

### 3.5. Wound Management

In our group, the wound management was generally effective, with no cases of skin necrosis or significant wound complications reported. There was one case of a superficial infection which was resolved, without further issues, with first-line empirical antibiotic therapy.

## 4. Discussion

The results of our study contribute to the ongoing debate about whether WALANT is truly necessary for outpatient hand surgeries. While WALANT offers notable benefits in some cases, particularly for longer or more complex procedures, our findings suggest that traditional LA-T remains a highly effective and safe option, especially for shorter surgeries such as carpal tunnel release, trigger finger release, and De Quervain’s tenosynovitis.

There is considerable evidence supporting the benefits of WALANT, particularly in terms of improving patient comfort during surgery. For instance, Saleh et al. [17] demonstrated that patients undergoing carpal tunnel decompression and trigger finger release with WALANT reported greater intraoperative comfort compared to those who underwent the same procedures using a tourniquet. The absence of a tourniquet avoids the associated pressure pain, which can become uncomfortable, especially during longer procedures.

The discrepancy between the findings in Saleh et al.’s study and our own may stem from several factors. First, in our study, we focused on shorter, routine procedures. For these surgeries, the use of a tourniquet for a brief period (around 12 min on average) likely caused minimal discomfort, particularly given that the surgeries were completed quickly. 

A systematic review by Olaiya et al. [18] further supported these findings, indicating that patients experienced less perioperative discomfort with WALANT due to the elimination of the tourniquet. However, despite the reduction in discomfort, the overall patient satisfaction between WALANT and LA-T was found to be comparable. This suggests that while WALANT may offer advantages in terms of comfort, it is not necessarily superior in terms of overall patient outcomes. Both techniques can lead to highly satisfying results, with the choice depending on individual circumstances and the nature of the surgery.

One of the key factors in determining whether LA-T is suitable is the patient’s tolerance of the tourniquet. Research shows that discomfort typically increases after 17 min of tourniquet use, which can be a limiting factor for longer surgeries [19]. However, in this study, where the tourniquet time was kept short, the pain associated with the tourniquet was minimal, with patients reporting an average VAS score of 2 out of 10. Immediate satisfaction was also high, with scores around 9.4 in the LA-T group compared to 9.6 in the WALANT group. This suggests that for shorter procedures, the tourniquet is well tolerated by most patients. Similarly, Gunasagaran et al. [19] found that while patients preferred surgeries without a tourniquet, the operative times between WALANT and LA-T were almost identical, indicating that the advantages of WALANT may be less significant for shorter-duration procedures.

Moreover, it must be remembered that WALANT requires a precise setting which is not easily guaranteed in every hospital, especially in short procedures like CTR. As at least 30 min are required after the injection, adequate pre-procedural room and personnel is required. In many environments, patients wait outside the OR where anesthesia cannot be administered beforehand, losing the “time” advantage of WALANT.

The possibility of using forearm placement for the tourniquet presents a valuable option for patients who experience significant discomfort or intolerance to the upper arm tourniquet. Lefebvre et al. [20] showed that forearm tourniquet placement allows for a longer inflation time, typically extending the tolerance period by an average of 4 min compared to upper arm placement. Additionally, forearm tourniquets are subjectively more comfortable, likely due to reduced pressure on larger nerve bundles and muscles, such as the biceps, which are more sensitive in the upper arm. For patients who are particularly intolerant to upper arm tourniquet pain, this alternative placement could significantly enhance their intraoperative comfort while still maintaining the necessary bloodless surgical field. The forearm’s smaller musculature and lower sensitivity make it a feasible choice, especially in procedures of moderate duration, potentially improving patient satisfaction without compromising the efficacy of the surgery.

While WALANT eliminates the discomfort caused by the tourniquet, it is not without its risks, particularly due to the use of epinephrine. Although complications are rare, they can be serious when they occur. For example, Zhang et al. [21] reported a case of fingertip gangrene leading to amputation after a trigger finger surgery performed with WALANT. Additionally, Zhu et al. [22] described a patient who developed prolonged ischemia after carpal tunnel release and trigger finger surgery, which was eventually reversed but left the patient with cold intolerance. These cases highlight the importance of carefully selecting patients for WALANT, particularly those with vascular conditions, as epinephrine-induced vasoconstriction can lead to severe complications. In WALANT, if the local anesthetic injection is not planned early enough prior to the incision, the optimal epinephrine vasoconstriction effect will not be obtained, McKee DE et al. established that waiting approximately 30 min after the injection of local anesthesia with epinephrine, as opposed to the traditionally taught 7 min, results in optimal vasoconstriction [23].

In our study, we did not observe any cases of digital necrosis, which is consistent with previous research demonstrating that WALANT, when used properly, is generally safe and achieves adequate hemostasis. Very few studies have documented complications specifically related to WALANT in trigger finger release. For example, Reynolds et al. [24] reported a 2.5% complication rate in 314 trigger finger surgeries performed with WALANT. Other studies have shown a wide range of complication rates for A1 pulley release surgeries, varying from 1% to as high as 43%, depending on the definitions used for complications and the methodologies employed.

The study conducted by Far-Riera [25] found a complication rate of 5.58%, with most complications being minor and managed conservatively. Additionally, the reoperation rate was low, i.e., at 1.5%. These results suggest that WALANT, while generally safe, still presents a risk of complications, though they are typically manageable.

It is also important to consider that the use of sedation, compared to local anesthesia, has been identified as a potential risk factor for developing complications. Sedation can add another layer of complexity to surgeries, which is why WALANT is particularly valuable in eliminating the need for it. WALANT allows surgeries to be performed under local anesthesia, avoiding the risks associated with sedation. However, for experienced surgeons performing shorter procedures, LA-T provides an effective alternative that minimizes these risks and still offers good patient outcomes.

While WALANT offers clear benefits for certain types of surgeries, especially those that require longer operative times or active patient involvement, it may not always be necessary for shorter, simpler outpatient hand surgeries. LA-T remains a practical, effective, and well-tolerated option, providing similar levels of patient satisfaction and safety. The choice between WALANT and LA-T should ultimately depend on the specific needs of the patient and the surgeon’s experience, ensuring that the most appropriate method is chosen for each case.

### Study Limitations

This study has several limitations that must be considered. First, the absence of a control group restricts our ability to directly compare the outcomes of WALANT with other anesthetic techniques, such as general anesthesia or regional blocks, thereby limiting our understanding of the relative advantages and drawbacks. Additionally, the study population was predominantly elderly, which may affect the generalizability of our findings to younger or healthier individuals. The variability in patient comorbidities and their potential impact on anesthetic tolerance were not controlled for, which could influence the observed pain levels and complications. Furthermore, our assessment of pain and discomfort relied solely on VAS scores, which are subjective and may not fully capture the range of patient experiences. Finally, the relatively small number of cases where the tourniquet had to be released due to discomfort limits our ability to draw robust conclusions about the frequency and predictors of this complication. Future studies with a more diverse patient population and a control group are needed to validate our findings and provide a clearer understanding of the risks and benefits associated with WALANT in outpatient hand surgery.

## 5. Conclusions

Contrary to some concerns, the tourniquet itself does not typically cause significant pain to the arm. However, it can increase discomfort during particularly lengthy procedures or when performed by less experienced, younger surgeons who may take longer to complete the surgery compared to their more experienced, older counterparts. For experienced surgeons, the tourniquet and local anesthesia technique is often sufficient and avoids the complications associated with epinephrine. In contrast, the WALANT technique can improve intraoperative comfort for patients when longer surgical times are anticipated or when the procedure is performed by less experienced surgeons due to the elimination of tourniquet-related discomfort. However, despite its benefits, we believe that WALANT may not be justified for short-duration outpatient hand surgeries when considering the risk–benefit ratio. 

## Figures and Tables

**Table 1:** Demographic data.

	Value
Total number of patients	300
Mean age	64.02 years old
Gender distribution	
Male	111 (37%)
Female	189 (63%)
Age group distribution	
<75 years old	225 (75%)
>75 years old	75 (25%)
<80 years old	256 (85.4%)
>80 years old	44 (14.86%)

**Table 2:** Surgery duration.

	Mean Duration	Range
CTR	9.87 min	6–12
Trigger finger	10.8 min	8–13
De Quervain	11.5 min	10–13
Combined surgeries	12 min	11–18

CTR = carpal tunnel release.

**Table 3:** Relationship between VAS score and age and surgery duration.

Group	VAS Score (Mean and Standard Deviation)	*p*-Value
<75 years old	3.21 1.26	*p*-value < 0.05
>75 years old	4.92 1.38	
<13 min	3.65 1.4	*p*-value = 0.69
>13 min	3.5 1.54	
<15 min	3.63 1.5	*p*-value < 0.05
>15 min	5.4 2.09	

## Data Availability

Data available on request to the corresponding author.
